# Faster juvenile growth promotes earlier sex change in a protandrous hermaphrodite (barramundi *Lates calcarifer*)

**DOI:** 10.1038/s41598-021-81727-1

**Published:** 2021-01-26

**Authors:** Brien H. Roberts, John R. Morrongiello, David L. Morgan, Alison J. King, Thor M. Saunders, David A. Crook

**Affiliations:** 1 Research Institute for the Environment and Livelihoods, Charles Darwin University, Darwin, NT Australia; 2 School of BioSciences, The University of Melbourne, Melbourne, VIC Australia; 3 Freshwater Fish Group & Fish Health Unit, Centre for Sustainable Aquatic Ecosystems, Harry Butler Institute, Murdoch University, Murdoch, Australia; 4 Centre for Freshwater Ecosystems, School of Life Sciences, La Trobe University, Albury-Wodonga, VIC Australia; 5 Department of Primary Industries and Fisheries, Fisheries Research, Berrimah, NT Australia

**Keywords:** Evolutionary ecology, Population dynamics

## Abstract

The relationship between growth and sexual maturation is central to understanding the dynamics of animal populations which exhibit indeterminate growth. In sequential hermaphrodites, which undergo post-maturation sex change, the size and age at which sex change occurs directly affects reproductive output and hence population productivity. However, these traits are often labile, and may be strongly influenced by heterogenous growth and mortality rates. We analysed otolith microstructure of a protandrous (i.e., male-to-female) fish (barramundi *Lates calcarifer*) to examine growth in relation to individual variation in the timing of sex change. Growth trajectories of individuals with contrasting life histories were examined to elucidate the direction and extent to which growth rate influences the size and age individuals change sex. Then, the relationships between growth rate, maturation schedules and asymptotic maximum size were explored to identify potential trade-offs between age at female maturity and growth potential. Rapid growth was strongly associated with decreased age at sex change, but this was not accompanied by a decrease in size at sex change. Individuals that were caught as large females grew faster than those caught as males, suggesting that fast-growing individuals ultimately obtain higher fitness and therefore make a disproportionate contribution to population fecundity. These results indicate that individual-level variation in maturation schedules is not reflective of trade-offs between growth and reproduction. Rather, we suggest that conditions experienced during the juvenile phase are likely to be a key determinant of post-maturation fitness. These findings highlight the vulnerability of sex-changing species to future environmental change and harvest.

## Introduction

Sequential hermaphroditism, where organisms undergo ontogenetic sex change, is a striking life history feature of a wide range of marine fishes, invertebrates (molluscs and crustaceans) and plants. Sex-changing species may breed initially as males before transitioning into females (protandry), or vice-versa (protogyny), with some species also capable of bi-directional sex change^1^. Among fishes, sex change is less common than gonochorism (fixed sexes) but is nonetheless taxonomically widespread across at least 41 teleost families (> 450 species), including species that support significant commercial fisheries (e.g., shads, barramundi, wrasses, groupers)^2,3^. Because the sexes are not evenly distributed throughout age and size classes in hermaphroditic species, extrinsic factors (e.g., hydrology, water temperature, etc.) that affect growth or survival may affect the population dynamics of sex-changing species in different ways to gonochoristic (i.e. fixed sex) species^4–6^. As such, understanding the extrinsic and intrinsic factors regulating sex change is an essential aspect of managing exploited populations of hermaphroditic species^5,7,8^.

Considerable research effort has been dedicated to examining the evolutionary mechanisms underpinning sequential hermaphroditism^9–14^. At the interspecific level, the ‘Size Advantage Hypothesis’^10^ predicts that sex change is favoured if the relationship between body size (or age) and individual fitness differs between the sexes. Protandry arises under scenarios where the relationship between body size/age and fitness is stronger for females than males. In such systems, large female body size is favoured due to the increased size facilitating higher fecundity, while small males are still able to successfully compete for mating opportunities alongside larger males. Alternatively, protogyny is favoured when large size enables dominant males to monopolise reproductive access to females. Sex allocation theory predicts that individuals should change sex when the reproductive value (i.e. expected future reproduction, weighted by the probability of survival to future ages) of the second sex exceeds that of the current sex^1,10,12^. In cases where social hierarchy governs mating opportunities, as with most protogynous fishes, sex change may be initiated in response to changes in hierarchy structure such as the death of the dominant male within a harem^11,13,15,16^. Where near-random mating occurs with respect to male body size (as typical of protandrous species), sex change may be triggered once a sufficient size or age is attained^17,18^.

Growth in most fishes is indeterminant and the size and age at sexual maturation is thus strongly influenced by growth rate and mortality risk^19,20^. The relationship between growth rate and the timing of sex change within and among species can, however, be flexible^5,17^. In some species, sex change may occur at a relatively consistent size or age, whilst in others considerable overlap is evident in the age and size distributions of males and females^5,21,22^. Frequently, a proportion of individuals skip spawning as the first sex or mature directly into the second sex (‘primary females/males’^23^). Other individuals delay sex change, despite attaining a size and age at which sex change may be expected^17,24^. Such life-history divergences among individuals may be shaped by a wide range of factors, such as differences in individual physiology or morphology, local environmental conditions, and intra-specific social interactions^25–27^.

The cues that affect the timing of sex change must be well understood to predict population-level responses to disturbances, such as fishing harvest and climate change^5^. An inherent property of sequentially hermaphroditic species is that reproductive success is higher in the second sex^12,28^, which makes them susceptible to anthropogenic disturbance via several mechanisms. First, size-selective fishing practices may disproportionally target the larger sex, resulting in increasingly skewed sex ratios and potentially reducing egg and sperm production in protandrous and protogynous species, respectively^5^. Second, subsequent compensatory declines in the length and age at which sex change occurs may have the effect of reducing population fecundity, despite partially offsetting skewed sex ratios^8,29^. Third, degradation of aquatic habitats and climate change may impact growth and mortality rates which, in turn, may alter the timing of sex change in sequentially hermaphroditic species.

The aim of the current study was to investigate the relationship between individual growth rate and the timing of sex change in the barramundi (or Asian sea bass) *Lates calcarifer.* Barramundi is a facultatively catadromous, protandrous fish that inhabits coastal and fresh waters throughout the Indo-West Pacific region, where it supports significant commercial, recreational and subsistence fisheries^30^. Barramundi have widely been considered to mature as males at 3–5 years before transitioning to females at 4–8 years, a process reportedly driven by age rather than size^31^. A small percentage of barramundi are primary females^31^, and some individuals skip spawning as males and reproduce for the first time as females^30^. Spawning coincides with spring tides during the monsoonal wet season in saline estuaries or associated coastal areas^32^. Females may spawn several times over the course of a breeding season, and males may fertilise the eggs of multiple females^33,34^. Barramundi typically form large aggregations on the spawning grounds, suggesting that mating occurs randomly throughout the population and is not restricted to social groups^34^. Growth rates are highly variable among systems, habitats, cohorts and individuals^32,35,36^, although the link between growth rates and maturation remains unclear.

We investigated the relationship between age-specific growth rates in barramundi and age-at-sex change and size-at-sex change. Additionally, relationships between juvenile growth rate and size-at-age were analysed to explore potential trade-offs between growth, age-at-sex change and adult body size. Growth rates were compared between groups of individuals with contrasting sex change schedules to examine the influence of previous growth rate on the timing of protandrous sex change. The results are discussed with regards to their potential implications on population fecundity and productivity.

## Materials and methods

### Study sites

Barramundi otoliths were collected between 2001 and 2004 from the Fitzroy river catchment in the wet-dry tropical region of northern Western Australia (Figure S1)^37^. The Fitzroy River flows through the western Kimberly region and drains into the Indian Ocean at King Sound (17° 33′ 12″ S, 123° 35′ 20″ E). Discharge is highly seasonal, with flows peaking during the monsoon (December–April), and then progressively decreasing during the dry season (May to November). During the dry season, the river is usually restricted to a series of isolated pools that are sustained by alluvial aquifers^37,38^.

### Growth proxy, fish collection and sample selection

Otolith microstructure was analysed to explore barramundi growth throughout ontogeny using annual growth increments as a proxy for somatic growth (see^39^). A total of 400 barramundi were collected using recreational (hook and line) and commercial (gill nets) fishing methods from a range of sites within estuarine and freshwater reaches of the river and associated tributaries, as well as from King Sound (see^40^). Total length (mm) and weight (g) were recorded and the sex of each fish was determined by gonad examination via dissection. One otolith from each fish was embedded in 2-part epoxy resin, sectioned transversely through the primordium and mounted on glass slides. The age of each fish was estimated by counting the number of annuli in each otolith section^41^. The second otolith from 158 of these fish was selected for growth analyses. Otolith sections were viewed under a dissecting microscope, photographed and the distances between annuli (pairs of translucent and opaque rings corresponding to wet and dry season growth, respectively) were measured (µm) using image analysis software (Leica Application Suite, v. 4.2). A transect along the proximal axis from the core to the outer edge was used to recreate the growth history of each fish.

### Back-calculation

Reconstruction of growth histories via otolith increment analysis explicitly assumes that otolith growth is proportional to somatic growth across the life history^42^. In our barramundi samples, regression analysis demonstrated the fish length ($L$)—otolith radius ($R$) relationship was influenced by fish age, with older fish tending to have larger otoliths for their size than younger fish i.e., Lea's phenomenon; see^43^. Therefore, otolith increments were converted to back-calculated fish growth (in mm) based on length at capture. Most back-calculation models implicitly assume a linear L-R relationship; however, regression analysis demonstrated the L-R relationship was best described by a second-order polynomial function in our barramundi samples (ΔAIC to 3rd order polynomial: 1.6; ΔAIC to linear: 3.9; Figure S2; Table S1). Therefore, a back-calculation model was developed using the Polynomial Scale Proportional Hypothesis, following the methods outlined by Vigliola and Meekan^44^:

1 $${a}_{0}+ {a}_{1}{L}_{ij}+ {a}_{2}{L}_{ij}^{2}= \frac{{R}_{ij}}{{R}_{cpt}} ({a}_{0}+ {a}_{1}{L}_{cpt }+ {a}_{2}{L}_{cpt }^{2})$$

where ${L}_{i}$ is the length of the *i*th fish at age *j*, ${R}_{cpt}$ is otolith radius at capture and ${L}_{cpt}$ is the total length-at-capture. ${a}_{0},$ ${a}_{1}$ and ${a}_{2}$ were estimated by fitting a linear model to the overall R-on-L quadratic relationship:

2 $$R= {a}_{0}+ {a}_{1}L+ {a}_{2}{L}^{2}$$

${L}_{i}$ in Eq. (1) was then solved via numeric optimisation, using the *Polyroot* function in Rstudio. Each annual growth estimate was assigned a growth year based on back-calculation from known date of capture.

### Life history classification

A substantial impediment to drawing comparisons between contrasting sex change schedules in our barramundi samples is that the precise ontogenetic timing that an individual transitioned from male to female is unknown. Fish harvested as old, large females may have transitioned several years prior, whilst individuals captured as young, small males may have imminently transitioned into females had they not been captured. To address this issue, analyses were focused on three separate comparisons between groups of individuals. Firstly, to examine individual variation in the age at which fish transitioned, growth trajectories were compared between barramundi that had matured as females prior to their 5th birthday (hereafter referred to as young sex-changers) versus individuals that remained males beyond age 5 (old sex-changers). Secondly, to examine individual variation in size at sex change, growth trajectories were compared between barramundi captured as females smaller than 850 mm (small sex changers), versus barramundi captured as males larger than 850 mm (large sex changers). These age and size classifications were selected to be close to the population average (i.e., length and age at which 50% of the population had become females) and to allow for enough individuals in each group to facilitate growth comparisons between life-history types (see Table 1). Thirdly, growth rates were compared between large (i.e., > 850 mm) females and small (i.e., < 850 mm) males. These two groups of individuals are assumed to be undertaking the same life-history strategy (i.e. normal progression from male to female) but were captured at different stages of ontogeny. Comparisons between these groups allows inferences as to whether individuals that ultimately attain large, female status are simply ‘regular’ fish that have survived to an old age, or alternatively, whether large size is facilitated by rapid juvenile growth. Because we compared groups of individuals harvested at different stages of ontogeny, we focused our analyses on the first three years of life, as this is the period for which all groups had sufficient overlapping growth data. This three-year period is also considered most relevant because among-individual variation in growth becomes less pronounced as barramundi grow older^31,36^.

**Table 1:** Details of barramundi samples used in this study, showing the number of individuals classified into the life history types of interest.

	Males			Females			Total
	Total	≥ 850 mm	≥ Age 5	Total	< 850 mm	< Age 5	
Age data	341	33	32	59	34	39	400
Growth data	115	27	29	43	23	25	158

Table shows the number of individuals per group for which age data were available, and the subset of individuals subjected to growth analyses.

### Statistical analysis

A Generalised Linear Model (GLM) with a binomial distribution (logit link function) was fitted to the proportion of females in each age class (1-year intervals) and size class (50 mm length intervals) to estimate the length and age at which 50% of the males in the population underwent sexual transition. The proportions in the GLM were weighted to account for heterogeneity in the underlying sample sizes^45^. To assess the relative importance of size and age in determining when individuals change sex, models were compared containing *age* and *length* fitted to barramundi *sex*.

Three different subsets of the barramundi growth data set were then explored: (i) young sex-changers and old sex-changers (to investigate the relationship between growth rate and the *age* that individuals changed sex); (ii) large sex-changers and small sex-changers (to investigate the relationship between growth rate and the *size* that individuals changed sex); and (iii) large females and small males (to test whether growth rate affects the likelihood of becoming a large, highly fecund female).

A mixed effects modelling framework was developed to investigate the relationship between growth trajectory (back-calculated *length-at-age*, mm) and *Sex-at-capture*, for each of the three subsets of growth data reflecting different life history comparisons. A series of models were developed using the *lme4* package^46^. These models contained different sets of intrinsic (individual) and extrinsic (environmental) predictor variables, and their interactions. A fixed *Age* effect was included to allow for declining growth rates with increasing age. A random intercept for *FishID* was included to account for repeated measures of increment data from individual fish, and to allow each fish to have higher or lower growth than the model intercept. A random *Age* slope for *FishID* (Age|FishID) was also included. To account for any persistent growth affects among individuals from a common year class, a *Cohort* random intercept was included. We also tested whether fitting a quadratic term for age (interacting with sex) to the optimum model improved model performance. To satisfy model assumptions, *Length-at-age* and *Age* were log-transformed, and the predictor variables were mean-centered to facilitate model convergence. Random effects structures and fixed effects structures were compared using restricted maximum likelihood estimation (REML), and maximum likelihood (ML), respectively. The relative support for each model was assessed using Akaike’s Information Criterion, adjusted for small sample sizes (AICc).

## Results

### Age and size distributions of males and females

**Figure 1:**
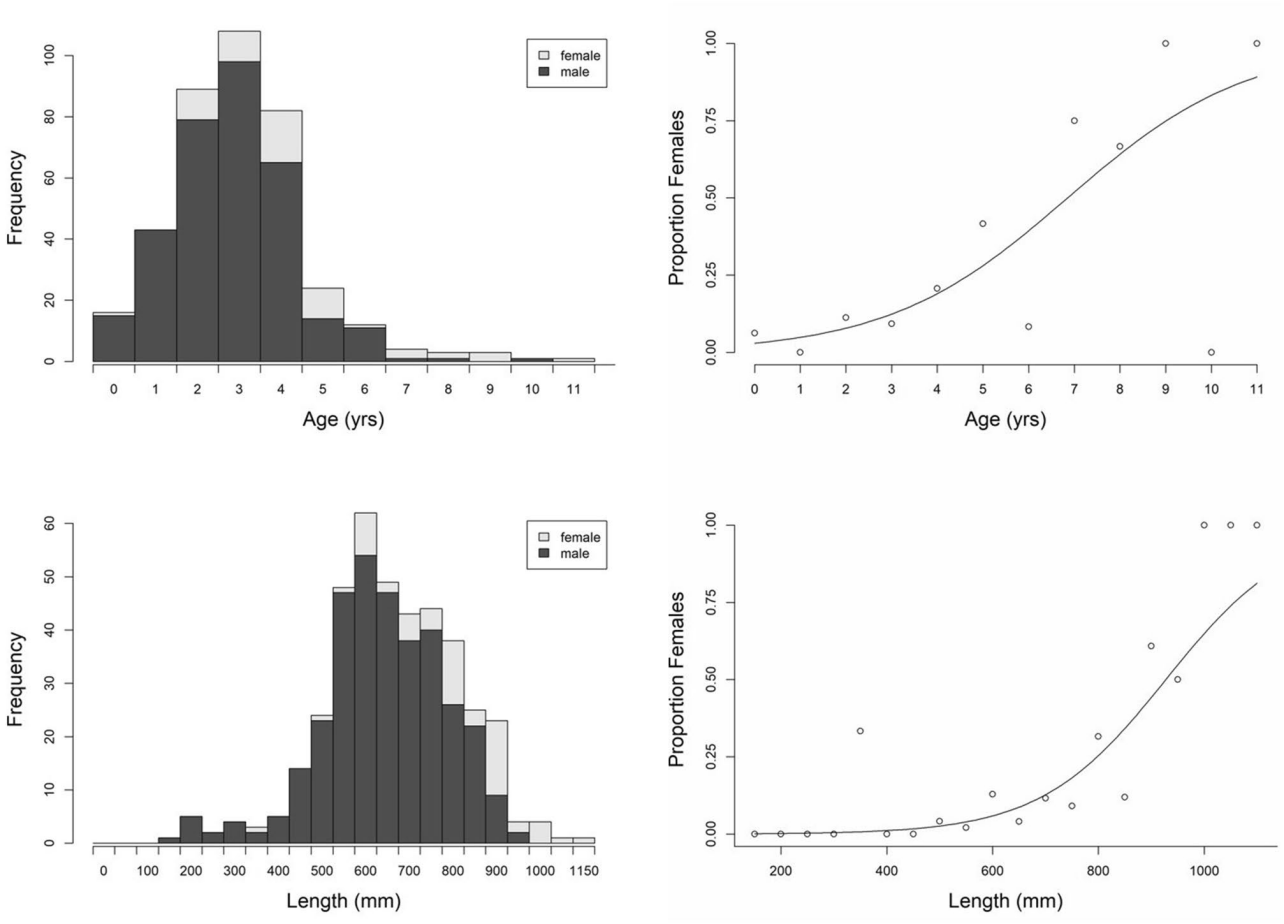
Stacked histograms (showing number of male and female barramundi in each size/age class) and weighted logistic curves (describing the proportion of females) as functions of age (grouped by 1-year intervals) and length (grouped by 50 mm intervals). n = 400 (341 males, 59 females).

Overall, the sex of barramundi was better predicted by length than age (ΔAIC to model containing *Age*: 1.7). There was considerable overlap with respect to the age and size distributions of male and female barramundi (Fig. 1). The oldest male was aged at 10 years (760 mm), and at least one ‘primary’ (i.e., not derived from a male) female (0 + years, 365 mm) was present in the dataset. L_50_ and A_50_ (the length and age at which 50% of captured fish were female) was 927 mm and 6.85 years, respectively.

### Growth differences between contrasting life histories

**Figure 2:**
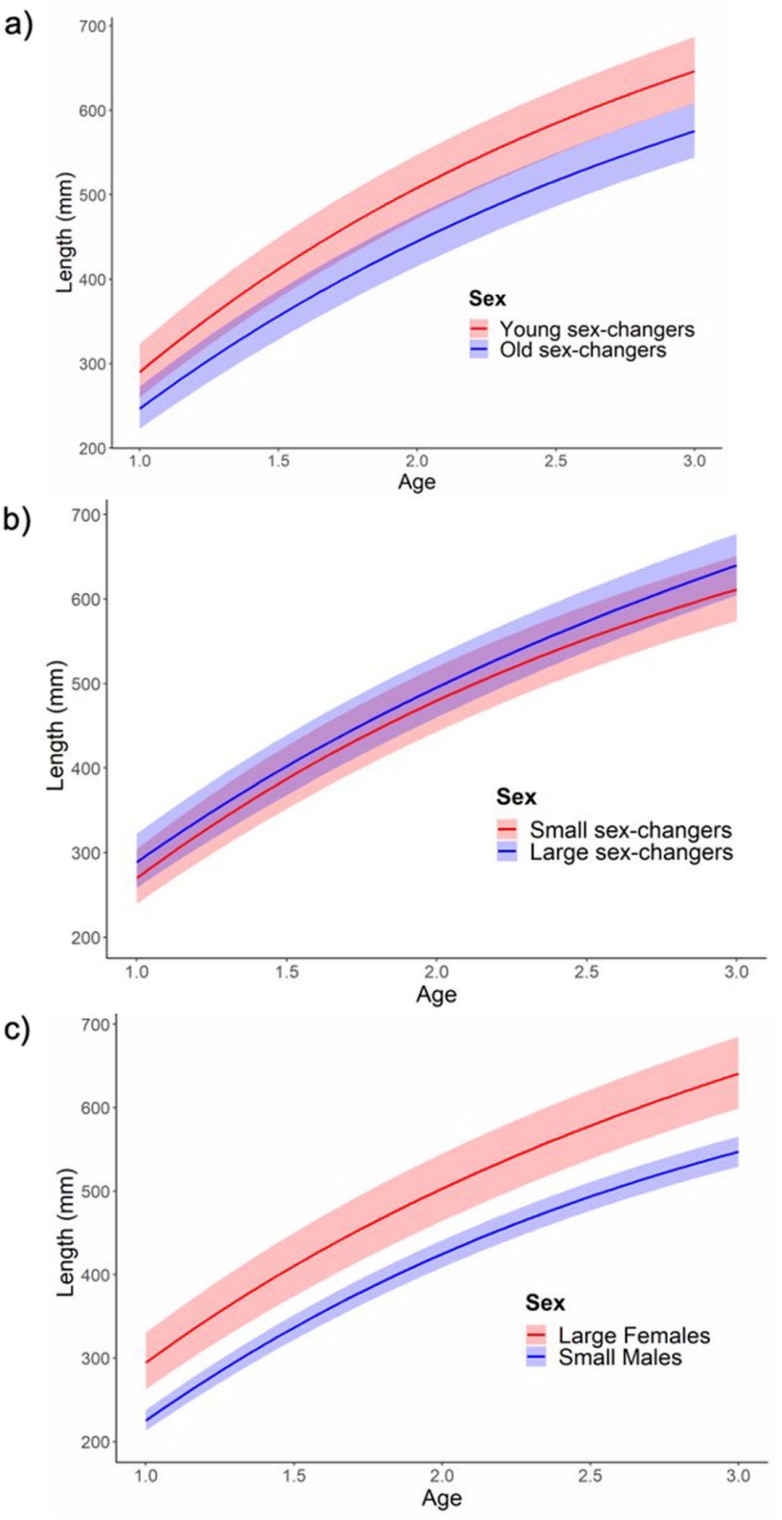
Linear mixed-effects growth curves illustrating differences in size-at-age between different life history types. Blue lines, males; red lines, females. (**a**) old sex-changers and young sex-changers (i.e., effect of growth rate on age of sex change); (**b**) large sex-changers and small sex-changers (i.e., effect of growth rate on size of sex change); (**c**) large females and small males. Shaded areas represent 95% confidence intervals.

Young female maturation was strongly associated with rapid growth during the juvenile phase (Fig. 2a, Table 2). Individuals that attained female status early had consistently fast growth rates across each of the first 3 years. However, growth rate was not strongly linked to variation in the size that barramundi underwent sexual transition (Fig. 2b). The largest individuals in the data set (i.e., those that were captured as large females) had substantially faster growth rates than those that were captured as small males (Fig. 2c). Results for the fixed and random effect model selection are provided in Table S2 and Table S3, respectively.

**Table 2:** Model parameter estimates with 95% CI describing fixed and random sources of growth variation in barramundi among each comparison of life-history types.

Data subset	Fixed effects				Random effects				
	Covariate	Estimate	Lower	Upper	Covariate	Estimate	Lower	Upper	Corr
Large sex-changers vs small sex changers	Intercept	6.101	6.000	6.217	FishID	0.176	0.139	0.220	
	Age	0.700	0.629	0.772	Age|FishID	0.168	0.137	0.208	− 0.820
	Sex male	0.053	− 0.076	0.174	1|Cohort	0.104	0.000	0.169	
	I (age)^2	− 0.217	− 0.285	− 0.156	Residual	0.03	0.024	0.038	
	Age × sex male	− 0.002	− 0.093	0.096					
	Sex male × I (age)^2	0.083	0.000	0.167					
Old sex-changers vs young sex-changers	Intercept	6.166	6.051	6.271	FishID	0.173	0.140	0.215	
	Age	0.685	0.617	0.749	Age|FishID	0.159	0.128	0.195	− 0.770
	Sex male	− 0.142	− 0.244	− 0.038	1|Cohort	0.115	0.000	0.190	
	I(Age)^2	− 0.224	− 0.293	− 0.156	Residual	0.038	0.030	0.043	
	Age × sex male	0.049	− 0.041	0.142					
	Sex male × I(Age)^2	0.040	− 0.049	0.132					
Large females and small males	Intercept	6.165	6.083	6.251	FishID	0.173	0.149	0.199	
	Age	0.676	0.610	0.739	Age|FishID	0.155	0.132	0.181	− 0.620
	Sex male	− 0.162	− 0.263	− 0.069	1|Cohort	0.003	0.000	0.108	
	I(Age)^2	− 0.159	− 0.232	− 0.091	Residual	0.037	0.031	0.042	
	Age × sex male	0.080	0.006	0.158					
	Sex male × I(Age)^2	− 0.106	− 0.186	− 0.020					

## Discussion

Considerable variation was evident in the timing of protandrous sex change in Fitzroy River barramundi. Overall, the timing of sex change was more closely related to an individual’s size than its age, thereby not supporting the assertion of Davis^31^ that barramundi sex change is primarily driven by age. Rapid growth was associated with female transition at younger ages, but did not strongly influence the size at which individuals changed sex. Individuals that were captured as large females had faster juvenile growth rates than those that were captured as smaller males, suggesting that rapid growth confers larger size-at-age throughout ontogeny. Notwithstanding potential trade-offs between growth and survival, this suggests that fast-growing individuals are more likely to attain female status sooner and have higher lifetime fecundity than slow-growing individuals. Our results therefore suggest that rapid growth may increase reproductive fitness by simultaneously increasing age-specific fecundity and the portion of ontogeny spent as a functional female.

The existence of extremely young, small females as well as large, old males among the samples may indicate that part of the barramundi population is gonochoristic. Indeed, primary females have been widely reported in a variety of protandrous species^17^, including barramundi^34^. In addition to primary females, some barramundi are known to skip spawning as males^30^, which may lead to increased growth and hence size in future spawning seasons^36^. In contrast, primary males are similarly widespread among protogynous fishes, a phenomenon which appears to be linked to population density (see^3^). Moreover, gonochoristic males (i.e. males that do not change sex) reportedly occur in the protandrous African threadfin *Polydactylus quadrifilis*^24^. Among our barramundi samples, however, the largest males tended to be relatively young; of the 35 large males, the oldest was 8 years of age and 870 mm in length. Given that females were aged up to 12 years, these large males presumably would have transitioned to females had they survived to a greater age. In contrast, the oldest males among the samples were invariably small. Such individuals may be gonochoristic in the sense that they never change sex, but this is likely because they failed to achieve a sufficient size, rather than an adaptive reproductive strategy.

Relatively large body size is a key determinant of reproductive value in fishes as it is linked to increased fecundity^47^, mating success^48^ and elevated survival^49^. Large body size is especially advantageous for sequential hermaphrodites, as fitness is inherently higher in the second, larger sex^12^. In Fitzroy River barramundi, fast growth was associated with younger female maturation and larger size-at-age throughout ontogeny. In this sense, the results suggest that fast growth may increase reproductive output. However, rapid growth may also be associated with traits which negatively affect survival (e.g., high-risk foraging behaviour or size-specific predation; see^50^). It is also plausible that size-selective harvest by commercial and recreational fishers may target larger individuals, and hence favour the survival of slower-growing individuals^42^. Since only individuals that survived until the time of capture are represented in our samples, any variation in mortality risk associated with growth rate in barramundi is not accounted for in our analyses.

**Figure 3:**
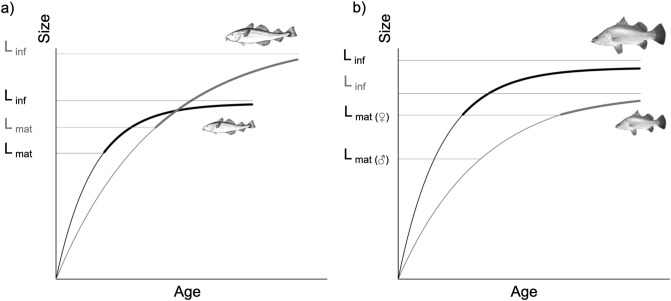
(**a**) Von Bertalanffy growth curves representing slow (grey line) and fast (black line) life histories in Atlantic cod. Thickened lines indicate length of maturity for the different life histories (modified from Audzijonyte et al.^66^). Trade-offs between growth and reproduction drive a continuum between slow (late maturation; high L-infinity) and fast (early maturation; low L-infinity) life histories, which are stable in an evolutionary sense in the absence of size-specific mortality. (**b**) In contrast, fast barramundi growth rates (black line) are linked to younger female maturation and larger size-at-age throughout the life history relative to slow growth rates (grey line).

The tendency for fast-growing fish to undergo female transition younger, but not smaller, than slow-growing individuals suggests that trade-offs between growth and reproduction do not play a major role in shaping individual variation in maturation schedules of barramundi (sensu^51^; Fig. 3). If such trade-offs were strongly influencing individual variation in sex change schedules, slow-growing individuals would be expected to ultimately attain larger sizes than those that initially grew fast and matured as females at young ages, as is the case for Atlantic cod (*Gadus morhua*; Fig. 3a). Fishing practices targeting large, fast-growing Atlantic cod have reportedly driven a rapid evolutionary shift towards fast life-histories^52^. Our results suggest that similar fishing effects would be unlikely to influence barramundi life-histories in the same way, but may nonetheless potentially have deleterious effects on recruitment. Coinciding with a period of increased fishing pressure, the proportion of female barramundi in the Fly river in Papua New Guinea reportedly declined from 27% in 1973 to 13% in 1978, which was not accompanied by a compensatory decline in the size at which males changed sex (Anon, cited in^31^). If large, fast-growing barramundi are disproportionally targeted by fisheries, it is likely that declines in female biomass may reduce population fecundity.

The largest barramundi in our samples were characterised by rapid growth throughout ontogeny. The apparent primacy of rapid growth with respect to fitness suggests that extrinsic factors may be a key driver of individual variation in sex change schedules (see^53^). For example, individuals with the highest fitness may simply be those that encountered favourable environmental conditions or lower rates of competition within local habitats during the early life history. It is also plausible that maturation schedules are influenced by phenotypic variation. For example, Luiz, et al.^54^ reported that individual variability in barramundi mouth gape was linked to body condition, where individuals with larger mouths tended to be in better condition. Presumably, these large-mouthed individuals would also have faster growth rates, suggesting that morphological characteristics may influence the timing of sex change. Our results could also be influenced by non-linear relationships between growth and maturation. For example, Alm^55^ suggested that Eurasian perch *Perca fluviatilis* with intermediate growth rates mature at large size, whereas fast- and slow-growing individuals mature at intermediate and small sizes respectively. Since our analyses did not include ‘intermediate’ sex-change schedules, such non-linear effects may not have been captured if they occur in barramundi.

Given the degree to which juvenile growth shapes size-at-age for subsequent life-history stages, these results suggest that conditions experienced during the early life history may strongly influence the timing of sex change (see^56^). Variation in maturity schedules may partially be shaped by the protracted spawning of barramundi, as individuals spawned at the beginning of the breeding season may get a ‘head start’ of several months on conspecifics spawned later^32,34^. During this period, young-of-year barramundi become increasingly piscivorous with increasing size (including cannabalism^57^), which may facilitate rapid increases in growth rate (see^58^) and drive extreme size heterogeneity within cohorts^59^. Thus, recruits that are spawned earlier in the breeding season, or encounter high quality habitats during the early life-history, may potentially obtain a substantial size advantage over those that are spawned later, and in turn might attain female status at a younger age.

Variation in sex change schedules may also be partially shaped by environmental heterogeneity. As is typical of riverine environments in the wet/dry tropics of northern Australia, flows in the Fitzroy system are highly seasonal, peaking during the monsoon season and progressively decreasing throughout the dry season. The duration and magnitude of wet season flows also varies considerably between years^38,60^, and years of high discharge have previously been related to barramundi recruitment^61,62^ and growth^35^. Our analyses do not link differences in individual sex-change regimes to specific cohorts or hydrological variables. However, given the scale of spatial and temporal fluctuation in habitat quality and quantity within tropical riverine systems^63^, including the Fitzroy River^38^, environmental factors appear likely to play an important role in shaping individual variation in growth and maturation schedules at scales beyond the scope of our analyses. Indeed, such life-history variation may enhance population resilience and stability, as diversified life-history types are widely considered to enable species to optimise recruitment in unpredictable environments (‘portfolio effect’^64^). Understanding how environmental variation—and river hydrology in particular—affects the expression of barramundi life history traits remains an important area for future research, especially considering the increasing demands for water resources and the predicted impacts of climate change in the region^65^.

In conclusion, this study demonstrates that the timing of female maturation in barramundi is strongly linked to juvenile growth rate. Given the link between growth and fitness, our results suggest that fast-growing fish may make a disproportionate contribution to population fecundity. If growth rates are impacted by selective fishing practices or degradation of aquatic habitats (e.g., river floodplains^63,65^), it is likely that the productivity of barramundi fisheries will be adversely affected. Our study therefore underscores the importance of information regarding relationships between growth rates and sexual maturation in fish, and how these relationships may be affected by future environmental change.

## Supplementary Information

Supplementary Information

## Data Availability

The dataset analysed in this study is available from the corresponding author on request.

## References

[1] Charnov EL (1982). The Theory of Sex Allocation.

[2] Pauly D (2007). Darwin's Fishes: An Encyclopedia of Ichthyology, Ecology, and Evolution.

[3] Kuwamura T, Sunobe T, Sakai Y, Kadota T, Sawada K (2020). Hermaphroditism in fishes: An annotated list of species, phylogeny, and mating system. Ichthyol. Res..

[4] Schultz ET, Warner RR (1991). Phenotypic plasticity in life-history traits of female Thalassoma bifasciatum (Pisces: Labridae): 2. Correlation of fecundity and growth rate in comparative studies. Environ. Biol. Fishes.

[5] Alonzo SH, Mangel M (2005). Sex-change rules, stock dynamics, and the performance of spawning-per-recruit measures in protogynous stocks. Fish. Bull..

[6] Hamilton SL (2007). Size-selective harvesting alters life histories of a temperate sex-changing fish. Ecol. Appl..

[7] Platten JR, Tibbetts IR, Sheaves MJ (2002). The influence of increased line-fishing mortality on the sex ratio and age of sex reversal of the venus tusk fish. J. Fish. Biol..

[8] Moore BR, Stapley JM, Williams AJ, Welch DJ (2017). Overexploitation causes profound demographic changes to the protandrous hermaphrodite king threadfin (*Polydactylus macrochir*) in Queensland’s Gulf of Carpentaria, Australia. Fish. Res..

[9] Bullough WS (1947). Hermaphroditism in the lower vertebrates. Nature.

[10] Ghiselin MT (1969). The evolution of hermaphroditism among animals. Q. Rev. Biol..

[11] Robertson D (1972). Social control of sex reversal in a coral-reef fish. Science.

[12] Warner RR, Robertson DR, Leigh EG (1975). Sex change and sexual selection. Science.

[13] Shapiro DY (1987). Differentiation and evolution of sex change in fishes. Bioscience.

[14] Avise JC, Mank JE (2009). Evolutionary perspectives on hermaphroditism in fishes. Sex. Dev..

[15] Mackie M (2003). Socially controlled sex-change in the half-moon grouper, *Epinephelus rivulatus*, at Ningaloo Reef, Western Australia. Coral Reefs.

[16] Liu M, Sadovy Y (2004). The influence of social factors on adult sex change and juvenile sexual differentiation in a diandric, protogynous epinepheline, *Cephalopholis boenak* (Pisces, Serranidae). J. Zool..

[17] Munday PL, Buston PM, Warner RR (2006). Diversity and flexibility of sex-change strategies in animals. Trends Ecol. Evol..

[18] Sunobe T, Sakaida S, Kuwamura T (2016). Random mating and protandrous sex change of the platycephalid fish *Thysanophrys celebica* (Platycephalidae). J. Ethol..

[19] Dieckmann U, Heino M (2007). Probabilistic maturation reaction norms: their history, strengths, and limitations. Mar. Ecol. Prog. Ser..

[20] Heino M, Dieckmann U, Godø OR (2002). Measuring probabilistic reaction norms for age and size at maturation. Evolution.

[21] Warner RR (1988). Sex change and the size-advantage model. Trends Ecol. Evol..

[22] Muñoz RC, Warner RR (2003). A new version of the size-advantage hypothesis for sex change: Incorporating sperm competition and size-fecundity skew. Am. Nat..

[23] Warner RR (1975). The reproductive biology of the protogynous hermaphrodite *Pimelometopon pulchrum* (Pisces: Labridae). Fish. Bull..

[24] Butler EC (2021). Do contemporary age-growth models overlook life-history complexities in protandrous fishes? A case study on the large protandrous polynemid, the giant African threadfin *Polydactylus quadrifilis*. Fish. Res..

[25] Ross RM (1990). The evolution of sex-change mechanisms in fishes. Environ. Biol. Fishes.

[26] Warner RR, Swearer SE (1991). Social control of sex change in the bluehead wrasse, *Thalassoma bifasciatum* (Pisces: Labridae). Biol. Bull..

[27] Kuwamura T, Nakashima Y (1998). New aspects of sex change among reef fishes: Recent studies in Japan. Environ. Biol. Fishes.

[28] Benvenuto C, Coscia I, Chopelet J, Sala-Bozano M, Mariani S (2017). Ecological and evolutionary consequences of alternative sex-change pathways in fish. Sci. Rep..

[29] Hawkins JP, Roberts CM (2004). Effects of fishing on sex-changing Caribbean parrotfishes. Biol. Conserv..

[30] Crook DA (2017). Use of otolith chemistry and acoustic telemetry to elucidate migratory contingents in barramundi *Lates calcarifer*. Mar. Freshw. Res..

[31] Davis T (1982). Maturity and sexuality in Barramundi, *Lates calcarifer* (Bloch), in the Northern Territory and south-eastern Gulf of Carpentaria. Mar. Freshw. Res..

[32] Davis T (1987). Biology of wildstock *Lates calcarifer* in northern Australia. Manag. Wild Cult. Sea Bass/Barramundi.

[33] Moore R (1982). Spawning and early life history of barramundi, *Lates calcarifer* (Bloch) Papua New Guinea. Mar. Freshw. Res..

[34] Garrett R (1987). Reproduction in Queensland barramundi (*Lates calcarifer*). Manag. Wild Cult. Sea Bass/Barramundi.

[35] Robins J (2006). Variable growth rates of the tropical estuarine fish barramundi *Lates calcarifer* (Bloch) under different freshwater flow conditions. J. Fish Biol..

[36] Roberts BH (2019). Migration to freshwater increases growth rates in a facultatively catadromous tropical fish. Oecologia.

[37] Morgan DL, Allen M, Bedford P, Horstman M (2004). Fish fauna of the Fitzroy River in the Kimberley region of Western Australia-including the Bunuba, Gooniyandi, Ngarinyin, Nyikina and Walmajarri aboriginal names. Records Western Aust. Museum.

[38] Lear KO (2019). Recruitment of a critically endangered sawfish into a riverine nursery depends on natural flow regimes. Sci. Rep..

[39] Morrongiello JR, Thresher RE, Smith DC (2012). Aquatic biochronologies and climate change. Nat. Clim. Change.

[40] 40Morgan, D. L. et al. Family Latidae, Giant Perches. In *A field guide to the freshwater fishes of the Kimberley* (eds J.J. Shelley, D. L. Morgan, M. P. Hammer, M. C. Le Feuvre, G. I. Moore, M. F. Gomon, M. G. Allen, & T. M Saunders) Murdoch University Print Production Team, 2018).

[41] Stuart I, McKillup S (2002). The use of sectioned otoliths to age barramundi (*Lates calcarifer*) (Bloch, 1790) [Centropomidae]. Hydrobiologia.

[42] Morrongiello JR, Thresher RE (2015). A statistical framework to explore ontogenetic growth variation among individuals and populations: A marine fish example. Ecol. Monogr..

[43] Campana SE (1990). How reliable are growth back-calculations based on otoliths?. Can. J. Fish. Aquat. Sci..

[44] Vigliola L, Meekan MG, Green BS, Mapstone BD, Carlos G, Begg GA (2009). The back-calculation of fish growth from otoliths. Tropical Fish Otoliths: Information for Assessment, Management and Ecology.

[45] Zuur, A. F., Hilbe, J. M. & Ieno, E. N. *A Beginner's Guide to GLM and GLMM with R: A Frequentist and Bayesian Perspective for Ecologists*. (Highland Statistics Limited, 2013).

[46] Bates D, Mächler M, Bolker B, Walker S (2015). Fitting linear mixed-effects models using lme4. J. Stat. Soft..

[47] Barneche DR, Robertson DR, White CR, Marshall DJ (2018). Fish reproductive-energy output increases disproportionately with body size. Science.

[48] Bisazza A, Marconato A (1988). Female mate choice, male-male competition and parental care in the river bullhead, *Cottus gobio* L. (Pisces, Cottidae). Anim. Behav..

[49] Perez KO, Munch SB (2010). Extreme selection on size in the early lives of fish. Evolution.

[50] Dibattista JD, Feldheim KA, Gruber SH, Hendry AP (2007). When bigger is not better: Selection against large size, high condition and fast growth in juvenile lemon sharks. J. Evol. Biol..

[51] Roff DA (1983). An allocation model of growth and reproduction in fish. Can. J. Fish. Aquat. Sci..

[52] Olsen EM (2004). Maturation trends indicative of rapid evolution preceded the collapse of northern cod. Nature.

[53] Collin R (1995). Sex, size, and position: a test of models predicting size at sex change in the protandrous gastropod *Crepidula fornicata*. Am. Nat..

[54] Luiz OJ (2019). Does a bigger mouth make you fatter? Linking intraspecific gape variability to body condition of a tropical predatory fish. Oecologia.

[55] Alm G (1959). Connection between maturity, size, and age in fishes. Inst. Freshw. Res. Rep..

[56] Walker S, Ryen C, McCormick M (2007). Rapid larval growth predisposes sex change and sexual size dimorphism in a protogynous hermaphrodite, *Parapercis snyderi* Jordan & Starks 1905. J. Fish Biol..

[57] Pusey B, Kennard MJ, Arthington AH (2004). Freshwater Fishes of North-Eastern Australia.

[58] Post DM (2003). Individual variation in the timing of ontogenetic niche shifts in largemouth bass. Ecology.

[59] Ribeiro FF, Qin JG (2013). Modelling size-dependent cannibalism in barramundi *Lates calcarifer*: Cannibalistic polyphenism and its implication to aquaculture. PLoS ONE.

[60] Douglas MM, Bunn SE, Davies PM (2005). River and wetland food webs in Australia's wetdry tropics: General principles and implications for management. Mar. Freshw. Res..

[61] Staunton-Smith J, Robins JB, Mayer DG, Sellin MJ, Halliday IA (2004). Does the quantity and timing of fresh water flowing into a dry tropical estuary affect year-class strength of barramundi (*Lates calcarifer*)?. Mar. Freshw. Res..

[62] Halliday I, Robins J, Mayer D, Staunton-Smith J, Sellin M (2010). Freshwater flows affect the year-class strength of Barramundi *Lates calcarifer* in the Fitzroy River estuary, Central Queensland. Proc. R. Soc. Queensl..

[63] Crook DA (2020). Tracking the resource pulse: Movement responses of fish to dynamic floodplain habitat in a tropical river. J. Anim. Ecol..

[64] Schindler, D. E. *et al.* Population diversity and the portfolio effect in an exploited species. *Nature* **465**, 609–612, http://www.nature.com/nature/journal/v465/n7298/suppinfo/nature09060_S1.html (2010). 10.1038/nature09060 20520713

[65] King AJ, Townsend SA, Douglas MM, Kennard MJ (2015). Implications of water extraction on the low-flow hydrology and ecology of tropical savannah rivers: An appraisal for northern Australia. Freshw. Sci..

[66] Audzijonyte AE (2016). Trends and management implications of human-induced life-history changes in marine ectotherms. Fish Fish..

