# Supplementary material for: Faster juvenile growth promotes earlier sex change in a protandrous hermaphrodite (barramundi Lates calcarifer)
Source: Sci Rep. 2021 Jan 26;11:2276. doi: 10.1038/s41598-021-81727-1 (PMC7838401; doi:10.1038/s41598-021-81727-1)
Supplement: Supplementary file 1 — Supplementary Information [file 41598_2021_81727_MOESM1_ESM.docx]

**Electronic Supplementary Material (ESM)**

**Faster juvenile growth promotes earlier sex change in a protandrous hermaphrodite (barramundi *Lates calcarifer*)**

Brien H. Roberts*^1^ · John R. Morrongiello^2^ · David L. Morgan^3^ · Alison J. King^1^ · Thor M. Saunders^4^ · David A. Crook^1^

^1^Research Institute for the Environment and Livelihoods, Charles Darwin University, Darwin, Northern Territory, Australia.

^2^School of BioSciences, The University of Melbourne, Victoria, Australia.

^3^Freshwater Fish Group & Fish Health Unit, Centre for Sustainable Aquatic Ecosystems, Harry Butler Institute, Murdoch University, Murdoch, Australia

^4^Fisheries Research, Department of Primary Industries and Fisheries, Berrimah, Northern Territory, Australia

*Corresponding author: email: [brien.roberts@cdu.edu.au](mailto:brien.roberts@cdu.edu.au); mobile: +61 488 298 406


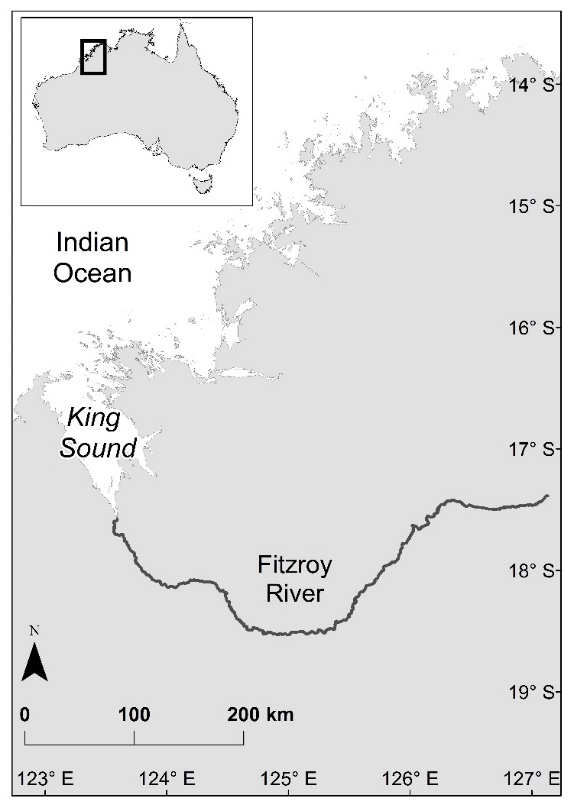


**Figure S1.** Map of the study region. Map produced using ArcMap 10.4 (ESRI, Redlands, Ca., <https://desktop.arcgis.com/en/arcmap/>).


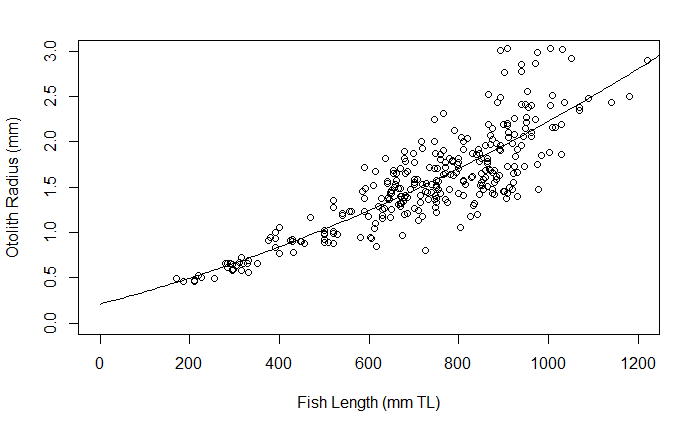


**Figure S2.** Regression for the relationship between otolith radius and fish length. Sample size: 305; R^2^: 0.716.

Table S1: Model selection results describing the otolith radius ~ fish length relationship used to develop the back-calculation model.

| Model | df | AIC |
| --- | --- | --- |
| Linear | 3 | 130.971 |
| **2nd order polynomial** | **4** | **127.068** |
| 3rd order polynomial | 5 | 128.685 |

Table S2: Results for the fixed effect model selection. At this stage, models included the optimum random effects structure of Age|FishID + 1|Cohort. The best overall models are highlighted in bold. Df; degrees of freedom; AICc: AIC corrected for small sample size; LL: log-likelihood.

| Data Subset | Model | df | AICc | LL |
| --- | --- | --- | --- | --- |
| Large sex-changers & small sex changers | Age | 7 | -198.9 | 106.8 |
|  | Age x Sex | 9 | -195.3 | 107.3 |
|  | **Age x Sex + Age^2^ x Sex** | **11** | **-235.1** | **129.5** |
|  |  |  |  |  |
|  |  |  |  |  |
| Old sex-changers & young sex-changers | Age | 7 | -188.6 | 101.7 |
|  | Age x Sex | 9 | -192.9 | 106.1 |
|  | **Age x Sex + Age^2^ x Sex** | **11** | **-235.6** | **129.7** |
|  |  |  |  |  |
|  |  |  |  |  |
| Large females & small males | Age | 7 | -310.7 | 162.6 |
|  | Age x Sex | 9 | -320.9 | 169.8 |
|  | **Age x Sex + Age^2^ x Sex** | **11** | **-420.2** | **221.6** |

Table S3. Model selection results for random effects structures of barramundi growth. Models included the maximal fixed effects structure ofr Age x Sex. The best overall models are highlighted in bold. Df; degrees of freedom; AICc: AIC corrected for small sample size; LL: log-likelihood.

| Subset | Model | df | AICc | LL |
| --- | --- | --- | --- | --- |
| Large sex-changers & small sex changers | 1\|FishID | 6 | -101.3 | 57 |
|  | Age\|FishID | 8 | -173 | 38.1 |
|  | **Age\|FishID + 1\|Cohort** | **9** | **-176.5** | **41** |
|  |  |  |  |  |
| Old sex-changers & young sex-changers | 1\|FishID | 6 | -117.5 | 65 |
|  | Age\|FishID | 8 | -171.6 | 94.3 |
|  | **Age\|FishID + 1\|Cohort** | **9** | **-173.5** | **96.3** |
|  |  |  |  |  |
| Large females & small males | 1\|FishID | 6 | -245.1 | 128.7 |
|  | Age\|FishID | 8 | -299.4 | 158 |
|  | **Age\|FishID + 1\|Cohort** | **9** | **-299.4** | **159** |
